# Online dilemma discussions as a method of enhancing moral reasoning among health and social care graduate students

**DOI:** 10.1007/s40889-022-00143-9

**Published:** 2022-06-01

**Authors:** Soile Juujärvi, Liisa Myyry

**Affiliations:** 1 Department of Master Programmes and Digital Education, Laurea University of Applied Sciences, Vantaa, Finland; 2 Centre for University Teaching and Learning, Faculty of Educational Sciences, University of Helsinki, Helsinki, Finland

**Keywords:** Continuing education, Dilemma discussion, Graduate, Moral development, Online learning, Professions

## Abstract

Dilemma discussions have been proven to be one of the most effective methods to enhance students’ moral reasoning in ethics education. Dilemma discussions are increasingly arranged online, but research on the topic has remained sparse, especially in the context of continuing professional education. The aim of the present paper was to develop a method of dilemma discussions for professional ethics. The method was based on asynchronous discussions in small groups. Health and social care students raised work-related dilemmas from their experiences and discussed them in terms of professional values, ethical guidelines and theories. Participants in this quasi-experimental study were 87 first-term graduate students at a Finnish university of applied sciences. Health and social care students in two consecutive ethics courses constituted two experiment groups, whereas health and social care students and business students in other programmes served as control groups. Students filled in a Defining Issues Test (DIT2) at the beginning of their studies and three months apart. Statically significant increase in moral reasoning was evidenced for experiment group 2, when discussion groups were purportedly composed to maximise differences in initial levels of moral reasoning. Findings suggest that online dilemma discussions can advance students’ moral reasoning development, especially when students’ exposure to higher-level arguments is ensured through complementary means, such as instructions, examples and plenary discussions. Online real-life dilemma discussions may also serve other important goals of ethics education, especially acquiring ethical concepts, and they can promote other components of ethical decision making: ethical sensitivity and motivation, and acquisition of implementation skills.

## Introduction

Reflective ethical decision making is a fundamental dimension of caring professions, such as nursing and social work, that aims to serve the wellbeing of other people. Ethical decision making can be defined as making a reasonable and responsible choice from among several alternatives in professional practice (Lechhasseur et al. 2018). It is supposed to be guided by legal and moral duties, and professional values that emphasise beneficence, human rights, equity and social justice (ICN 2012; ISFW 2018). Therefore, professional schools should prepare students to deal with ethical issues, requiring moral knowledge and capacities in moral problem solving. Ethical decision making can be viewed as a multi-determined process involving moral sensitivity (ability to recognise and interpret moral issues), moral judgment (judging which action is ethically right among various alternatives), moral motivation (prioritising moral values over other values) and moral character (perseverance, implementing skills) (Rest 1994). According to the current cognitive developmental approach in moral psychology, all elements are needed to accomplish ethically valid actions; but nevertheless, moral judgment, including the processes of problem solving, remains the most critical element, especially when dealing with complex ethical dilemmas (Rest et al. 1999).

Due to advanced technologies, ethics courses are nowadays often arranged online, and the Covid-19 pandemic has accelerated this trend. Online ethics education, as online higher education in general, relies on group discussions. Discussion in small groups is expected to advance critical thinking and analysis skills through collective inquiry into the specific contents (Stone et al. 2017). Group discussions of controversial moral dilemmas, called dilemma discussions, have been established as one of the effective methods in enhancing moral thinking (King and Mayhew 2002; Schlaefli et al. 1985). In classic dilemma discussions, teachers select complex dilemmas for the stimulus material, set up and lead group discussions. Dilemma discussions focus on problem solving that expose students to different points of view and involve activities, such as challenging another’s thinking, re-examining assumptions, building lines of argument and responding to counterargument (Schlaefli et al. 1985). The method of dilemma discussion has also increasingly been implemented in online ethics education, yielding inconclusive findings (Hedayati-Mehdiabadi et al. 2020; Huschle 2013; Joiner and Jones 2003; Özçinar 2015). To our knowledge, there are very few studies concerning online dilemma discussions in professional schools (Cain and Smith, 2009; Huschle 2013; Juujärvi and Pesso 2008), especially in graduate education (for a review of group discussions, see Stone et al. 2017) and in working life contexts (Roche and Thoma 2017). Thus, there is a need to scrutinise the effect of online interventions on ethical thinking.

This study aims to address the above-mentioned research gap by planning and carrying out an intervention study in a graduate programme for health and social care practitioners. The study was conducted as a part of the ethics course based on the blended learning approach (Graham, 2012) that combined classroom teaching and asynchronous online dilemma discussions. The ethics course was designed according to the principles of the cognitive-developmental approach to moral development (cf. Bebeau 1994) and aimed to address the specific needs of adult learners with a wealth of professional experience.

In this study, we were particularly interested in whether students’ real-life dilemmas were appropriate as stimuli for moral reasoning. According to the recent model of integrative pedagogy (Tynjälä et al. 2020), adult learning can be advanced through progressive problem solving that integrates different elements of expertise knowledge, especially practical and conceptual knowledge. In our case, student experiences of diverse ethical issues represent practical knowledge, whereas professional values and ethical guidelines, and influential moral theories, represent conceptual knowledge. Compared with undergraduate education, graduate education should prepare students to develop more extensive and deep analyses of ethical issues, due to their rich experiences and the complexity of background materials (Stone et al. 2017), including ethical theories, guidelines, reports, media debates and so forth. Furthermore, ethical theories may suggest contradicting solutions, and students need to learn to apply them in unique situations (Fletcher 1966).

### Moral reasoning development in professions

The method of dilemma discussion has its roots in the cognitive developmental approach to moral development (Kohlberg 1984), according to which development means stepwise improvements in moral thinking. Progress in moral judgment can be described through developmental phases, denoting increasingly complex modes of co-operation and improved understanding of moral concepts, especially fairness and justice. According to the reformulation of Kohlberg’s theory (Rest et al. 1999), progress in moral reasoning, from adolescence to adulthood, is best described by three successive schemas, that is, cognitive structures residing in a long-term memory and providing a general conceptual bedrock for interpreting and solving moral issues (Narváez and Bock 2002). All these schemas are relevant to professional life, explained as follows.

*The personal interest schema* focuses on advancing interests of self and those of significant others in brief exchanges and negotiated co-operation. Professionals are inclined to live up to role expectations and keep promises based on mutual agreements and advance benefits of their organisations, but their moral duties do not extend beyond interpersonal relationships. The personal interest schema gives way to *the maintaining norms schema* when individuals start to grasp the need for morally valid co-operation among strangers within society. Rules, laws and hierarchies are understood as establishments of social order, and they are morally binding for their own sake. Professionals are inclined to fulfil agreed-upon duties and view themselves and co-workers as representatives of the professional communities and service systems. In ethical decision making, it is right to follow duties, laws and codes of ethics, and to contribute to the community, institution or society in order to advance the common good. *The postconventional schema* emerges when the individual realises that laws and social practices are socially constructed and can be biased against certain groups and individuals. They are, therefore, open to rational critique and can be challenged by new evidence with the respect of due process and objective impartiality. The individual has given up moral relativism and they are committed to shared moral values. In ethical decision making, professionals are able to question harmful and unethical conventions and adopt the perspectives of each party or person involved (Narváez and Bock 2002; Juujärvi and Helkama 2020).

To summarise, progress in moral reasoning means a gradual shift from preference of the personal interest schema towards the maintaining norms schema and finally the postconventional schema, while previous schemas remain available (Rest et al. 1999). Giving up the use of the personal interest schema is especially critical for professionals because ethical decision making cannot be based on advancing egoistic interests (Thoma et al. 2008). The maintaining norms and postconventional schemas make professionals capable of conceptualising ethical dilemmas beyond personal relationships and are, therefore, a prerequisite for advanced ethical decision making concerning strangers and having far-reaching consequences for other people not involved in the immediate situation. An increase on postconventional schema is an especially important goal of ethics education, because it equips professionals with critical understanding of prevailing social practices that can compromise ethical values and thus prepare to act in the best interests of clients even under social pressure or organisational constraints. This is crucial for nurses and social care workers who are morally responsible to their patients and clients, but simultaneously accountable for their organisations. While norms and procedures are generally beneficial for professional practices, they are not ends as themselves but should be evaluated in terms of their contribution to patients’ wellbeing (de Casterlé et al. 2008). Based on a meta-analysis of 1592 nurses across four countries, de Casterlé et al. concluded that “nurses tend to reason in a conformist way in daily ethical dilemmas, being guided by conventional workplace rules and norms, rather than using creativity and critical reflection” (p. 548) and recommended to seek ways to promote nurses’ ethical development towards a postconventional practice enabling the realisation of patient-centred values.

Neo-Kohlbergian researchers (Bebeau and Thoma 1999; Thoma 2006, 2014; Thoma et al. 2008) have further advanced moral judgment as a multi-layered construct for ethical decision making of professionals, and consequently, proposed three levels for its functioning: schemas, intermediate concepts and codes of conduct. Codes are the most prescriptive and require the least interpretation, because correct actions in particular situations are dictated, and the most difficult aspect is to decide whether a situation at hand is concerned with the code (Thoma 2006). Intermediate concepts represent moral concerns that are often connected to the central tasks and responsibilities of the professions. They are used to communicate ethical standards of the professions, such as informed consent or patient confidentiality, and they are applicable to a broad range of situations requiring interpretation (Bebeau and Thoma 1999). Schemas are the most general and the least content-specific, and they provide a bedrock that is used as a default system and are triggered when codes and intermediate concepts fail to resolve moral issues (Thoma 2006).

All levels of moral judgment should operate in ethical decision making. Students’ understanding of intermediate concepts, such as values and ethical principles, are shaped by the bedrock schema (Thoma et al. 2008); therefore, dilemma discussions reveal a diversity of interpretations. The rationale of dilemma discussions is based on the idea that students are exposed to hearing and adopting more advanced arguments from their peers that supports progress towards higher schemas. As the second purpose, students have to gain moral knowledge and argumentation skills in order to able communicate their moral opinions and ethical convictions to other people (Duckett and Ryden 1994; McLeod-Sordjan 2014). Particular moral theories are consistent with the post-conventional schema; therefore, examination of ethical issues through them may advance students’ moral reasoning.

### Dilemma discussions

What is the role of education in moral reasoning development? It is maintained that higher education per se promotes moral reasoning development through general intellectual stimulation and advanced role-taking opportunities, but exposure to effective ethics education can also accelerate development (Rest 1994). Consistent with this claim, it has been found that higher education also tends to promote moral reasoning development (Mayhew et al. 2015; Myyry et al. 2013; O'Flaherty and Gleeson 2014) in nursing and social services programmes (e.g., Duckett and Ryden 1994; Juujärvi 2006) even though progress in general seems to remain modest (see Molloy et al. 2016; Numminen and Leino-Kilpi 2007). In addition to dilemma discussions (Boss 1994; Bunch 2005; Keefer and Ashley 2001), a variety of methods have proven effective, including courses focusing on raising the awareness of diversity (Hurtado et al. 2012; Parker et al. 2016) or others in need (Lies et al. 2012), and interactive methods (Auger and Gee 2016; Saat et al. 2012).

The quality of dilemmas has an important role in sustaining and nurturing discussion. They can be selected by teachers or students, both alternatives having advantages and disadvantages. Dilemmas promoting moral reasoning should be complex enough to encourage students to reason about moral issues from broader societal perspectives (Mayhew and King 2008) and can be proposed by teachers. In contrast, authentic experiences proposed by students often present diverse ethical issues that are concerned with the practical consequences of decision making for self and others. (Banks and Williams 2005; Juujärvi et al. 2020). While these dilemmas can be emotionally engaging, they do not necessarily expose students to broader societal perspectives. In our view, the use of real-life dilemmas is nevertheless a better alternative, because they serve the integration of lived experiences with moral concepts and promote other components of ethical decision making: ethical sensitivity and motivation, and implementation skills.

### Online dilemma discussions

Online group discussions can take diverse forms. They can be synchronous, asynchronous, text-based, audio, audio-visual and combinations of these (Stone et al. 2017). In synchronous online discussions, participants are online simultaneously, i.e. they are time dependent but independent of place. Asynchronous online discussions are both time and place independent because participants can participate at their own convenience. Moreover, asynchronous discussions allow participants to use as much time they like to think and write their response (Jonassen and Kwon 2001). Online discussions can furthermore be totally computer-mediated while participants interact only through different electronic communication tools (Özçinar 2015), such as e-learning platforms or video conferencing systems. They also can be embedded in the blended learning design that integrates online and classroom learning environments (Graham 2012; Spanjers et al. 2015). In this paper, we focus on asynchronous, text-based discussions, which is obviously the most usual mode of online group discussions.

Stone et al. (2017) compared asynchronous online and in-person group discussions. The distinguishing features of in-person settings are physical proximity and oral communication that provide non-verbal and paralinguistic cues lacking in online settings. This makes face-to-face conversations lively, but simultaneously challenging for the instructors, especially in big groups. Their responses to the raised topics can be delayed, and they can have difficulties constraining dominating students and encouraging shy ones. In asynchronous online settings, instructors can give constructive feedback more precisely, but their responses can nevertheless be delayed. Asynchronous online discussions can better engage socially reserved students when the absence of nonverbal clues may lessen inhibitions in responding critically to others (Cain and Smith 2009). Stone et al. (2017) conclude that both settings provide instructors with the possibility to be immersed in discussions and redirect discussion topics and threads, and the main challenge for both is to promote and sustain collaborative critical inquiry.

The expected benefit of asynchronous online discussions is that they sustain reflective inquiry, because they allow students to prepare their contributions before sharing; they can develop well-reasoned arguments with the expanded time. Effective discussions increase critical thinking and knowledge construction (for a review, see Putman et al. 2012). Cain and Smith (2009) noted that online discussions afforded all participants time to reflect and respond during discussions, making arguments available to others over long periods. In a more recent study, Hedayati-Mehdiabadi et al. (2020) observed that asynchronous online discussions promoted respondents’ ethical thinking by providing new insights and adding new perspectives to ethical issues. In particular, younger and inexperienced students seem to benefit from face-to-face teaching, whereas experienced college students performed as well in face-to-face and online settings (Huschle 2013).

Even though dilemma discussions should be based on students’ exchanges, multiple tasks of the instructor are critical for successful discussions. First, the instructor is responsible for creating supportive and safe learning environments, which is a precondition for engaging students in thought-provoking discussions and critical reflection, fostering growth in moral reasoning (Mayhew and King 2008). Merely sharing different opinions is not enough to elicit changes in moral thinking; peer students need to learn to challenge each other in constructive ways that can be exemplified by the instructor. Second, the instructor needs to make prompt interventions into discussions, in order to help students adopt wider societal perspectives on the issue at hand. Last but not least, the instructor has to plan detailed guidelines for discussions. To enhance the quality of the discussion, it is worthwhile to instruct students to write well-reasoned arguments (Bebeau 2002). The instructor also has to be familiarised with the software and be prepared to assist in solving technical problems.

Finally, what is a required time for discussions to enhance growth in moral reasoning? Past studies indicate 12 hours for face-to-face discussions among dentist students (Bebeau 2002), over 20 hours among medical students (Self et al. 1998) and over 30 hours among divinity students (Bunch 2005) although lower numbers have also been reported (Schlaefli et al. 1985). In the era of online learning, the calculation of time does not appear relevant, because it allows flexible learning arrangements and multitasking. The length of the discussion might be more important than amount of spent time because the change in thinking patterns is typically slow; discussions over 12 weeks are recommended (Schlaefli et al. 1985). In the case of graduate students, it is important that they are able raise meaningful topics from their work contexts and monitor their development over the course.

To wrap up the previous theoretical viewpoints, it can be assumed that online dilemma discussions promote students’ moral reasoning. Therefore, we hypothesise that graduate students participating in the online dilemma intervention will show growth in moral reasoning, whereas students not participating will stay constant in moral reasoning.

## Method

The intervention study was conducted in 2016 to 2018 as part of the research project aiming at investigating current competence needs of social and health care workers in Finland. Participants were 87 first-term students in three graduate programmes at the university of applied sciences in southern Finland participating in the project. The study employed a quasi-experimental design with two experiment and two control groups. The study plan was approved by the ethics committee of Federal Universities of Applied Sciences. The first author worked as a teacher at the course of ethics for intervention groups. The research data were collected separate from other learning procedures at the beginning of the course and within two weeks after its ending. It was emphasised that participation was voluntary and would not affect grades in any way. The participants undersigned informed consent.

Students at the two successive obligatory courses of healthcare and social welfare ethics (in 2016 and 2017) constituted two experimental groups. There were no classroom ethics courses available to serve as control groups, and therefore, students at the two other programmes were recruited for this purpose. The first control group involved first-term business students in 2016 and 2017, and the second one involved first-term social and healthcare students in 2018 (for participants’ backgrounds, see Table 1). To increase participation, students in all groups received time to complete pre-tests during regular class times. Post-tests were sent through emails with two reminders to all groups after twelve weeks, and students took them on their leisure time. Finally, students who completed both pre-tests and post-tests were validated as participants (for final participation rates, see Table 1).

**Table 1:** Background variables

	Experiment 1	Experiment 2	Control 1	Control 2
	*(n* = 22)	*(n* = 23)	(*n* = 18)	*(n* = 24)
Women % (*n*)	96%	87%	83%	96%
	(21)	(20)	(15)	(24)
Age *M*	36.4	38.0	35.1	40.0
(*SD)*	(7.6)	(7.4)	(5.4)	(7.6)
Work years *M* (*SD*)	9.6	9.2	8.8	13.7
	(6.3)	(3.7)	(4.5)	(6.0)
Professional backgrounds % (*n*)	Nursing: 50% (11) Social work: 32% (7)	Nursing: 43% (10) Social work: 57% (13)	Business: 78% (14)	Nursing: 21% (5) Social work: 71% (17)
	Other: 18% (4)		Other: 22% (4)	Other:8% (2)
Participation rate^1^	79%	86%	32%	83%

^1^Percentage of invited students who returned both pre- and posttest

The Defining Issues Test is (DIT) a well-established and widely used instrument measuring moral reasoning development in intervention studies (Thoma 2014). The revised version of DIT (DIT-2) was used to measure moral reasoning on the pre- and post-test. The short form of the DIT-2 presents three dilemmas to respondents, followed by the question about what the protagonist should do. Then respondents are asked to assess 12 arguments representing different moral schemas in terms of importance and rank the four most important ones for decision. Based on the calculation of rankings, the DIT-2 yields scores on personal interests schema (PIS), the maintaining norms schema (MNS) and the postconventional schema (PCS, also called P score). The P score is based on the participant’s ranking and prioritizing of post-conventional items. The DIT-2 also yields the N2 score that is a modified P score, representing the extent to which post-conventional items are prioritized, but adjusting the P-score based on the respondents’ ability to discriminate between P items and lower stage items; in other words, to give the PIS items lower rankings (Rest et al. 1999; Roche and Thoma 2017; Thoma and Dong 2014). The N2 can be recommended for studies with adult populations, because it reflects an individual’s increased understanding of a system of fairness that serves public good (O'Flaherty and Gleeson 2014; Thoma 2006).

The obtained data were processed with the SPSS software and sent to the Centre for the Study of Ethical Development at the University of Alabama, which scored and returned the data with multiple indices. The data was further analysed with appropriate statistical methods, using SPSS versio. In analyses, the significance level of 0.05 (*p* < .05) was considered statistically significant.

### Intervention

Online discussions were a part of the blended learning approach, which combined classroom teaching of 24 hours and asynchronous dilemma discussions. Classroom teaching focused on ethical guidelines, theories of moral philosophy, including the Kantian theory, utilitarianism, social justice approach, theories of moral psychology, including the ethics of care and justice, and contemporary ethical issues. In the blended learning approach, face-to-face encounters enable brainstorming and rapid communication about complex issues through lecturing or other means, whereas digital environments enable distant communication (Graham 2012; Spanjers et al. 2015). Accordingly, classroom learning involved lectures and group work, and online learning involved dilemma discussions accompanied by information search on the internet. The course was finalised by a written assignment through which students analysed their real-life dilemma in detail according to the instructions. The procedure was similar in both experiment groups, whereas the control groups participated in regular courses and did not receive any ethics-related treatment.

Online discussion groups involved four to five students. The students were informed that instructors would take part in the discussion, but the main responsibility for advancing the discussion would be theirs. In experiment 1, members for each group were selected to maximise diversity in their professional backgrounds, mixing nursing and social services students. In experiment 2, students’ scores on the pre-test were added as a criterion for composition of the groups, in order to maximise differences in moral reasoning levels within the group. Thus, the groups were planned to represent a mixture of occupations and moral schemas. The students were not aware of the specific criteria for the group compositions.

Online discussions were initiated by a hypothetical dilemma dealing with the issue of abortion. The aim of this dilemma was to practise online discussion to make students comfortable with the format and allow time to create useful rules and practices for the group. After the exercise discussion, the groups were instructed to establish rules and a timetable for their work. Discussion of each real-life dilemma was initiated by writing a starting message into a thread, each dilemma having threads of their own. The instruction for starting a discussion was as follows.

Describe a situation in working life that puzzled you and you were not sure what the right thing to do was. What issues caused you a problem in that situation and why? How did you act in that situation? Discuss your case in the online group discussion group. Consider the situation from your viewpoint and the viewpoint of other people involved in the situation. What issues should be taken into consideration? What would have been the right thing to do? Reflect on the case by referring to the professional ethics guidelines, personal and professional values, and ethical theories. Search for relevant knowledge to support your decision making.

Discussions were scheduled to last 10 weeks; after that, students needed to complete their assignments within three weeks. In both groups, one group out of seven encountered difficulties in establishing a discussion because of passive or quitting members. Students delivered 32.5 (SD = 19.1, group 1) and 37.4 (SD = 18.5, group 2) messages on average. The difference between intervention groups was not however statistically significant, *t*(1, 44) = −0.77, *p* = 0.45**.** The number of the delivered messages ranged remarkably: from 6 to 96 messages among students, and from 36 to 257 messages among discussion groups, respectively. The content of messages was varied, including opinion exchange, socio-emotional messages and links to the sources of information; and against the instructions, some students also wrote long essays. Instructors summarised viewpoints, asked critical questions, encouraged participation, and responded to practical questions.

## Results

The means and standard deviations of the DIT scores are reported in Table 2. According to one-way analyses of variance, groups’ personal interest, maintaining norms and postconventional scores on the pre-test were not different from each other: *F*(3, 85) = 0.711 for the personal interest score*, F*(3, 85) =1.315 for the maintaining norms score, *F*(3, 85) =1.45 for the post-conventional score, and *F*(3, 85) = 0.048 for the N2 score, respectively, all *ns*. To test whether the scores of the experiment and control groups have been affected by the intervention, we conducted a series of repeated analysis of variance for each score. Main effects of intervention were significant neither for the personal interest score, *F*(1, 83) = 0.92, nor the maintaining norms score, *F*(1, 83) = 0.22, respectively, both *ns,* and there were no significant intervention X group interaction effects: *F*(3, 83) = 0.73 for the personal interest score, and *F*(3, 83) = 1.90 for the maintaining norms score, respectively, both *ns*. Furthermore, main effects of intervention were significant neither for the P score, *F*(1, 83) = 0.02, nor the N2 score, *F*(1, 83) = 0.97, both *ns.* However, intervention X group interaction effects were significant: *F*(3, 83) = 4.27, *p* = 0.007, η = 0.13 for the P score, and *F*(3, 83) = 5.53, *p* = 0.002, η = 0.17 for the N2 score, respectively. The contrast comparisons with the Bonferroni adjustment revealed that for experiment group 2, the N2 score increased significantly from the pre-test to the post-test (*p* < 0.05), whereas the increase on the P score did not reach significance, *p* = .084.

**Table 2:** Means and standard deviations of the DIT scores and change on the P score and N2 score in experiment and the control groups

	Experiment 1	Experiment 2	Control 1	Control 2	Total
	*M* (*SD*)	M (*SD*)	M (*SD*)	M (*SD*)	M (SD)
Pre_PIS	25.30 (12.87)	19.42 (14.69)	21.48 (17.34)	24.44 (17.71)	22.72 (15.64)
Post_PIS	25.00 (13.12)	18.26 (12.74)	25.74 (20.67)	27.36 (18.20)	24.02 (16.42)
Pre_MNS	27.88 (16.08)	33.19 (11.52)	32.78 (13.00)	26.94 (13.04)	30.04 (13.57)
Post_MNS	30.75 (11.90)	29.13 (12.56)	38.15 (15.05)	25.55 (11.78)	30.42 (13.28)
Pre_PostC	41.67 (15.07)	38.84 (17.31)	40.18 (12.81)	42.08 (15.03)	40.73 (15.06)
Post_PostC	39.70 (13.21)	45.51 (15.09)	30.55 (13.44)	40.83 (17.23)	39.65 (15.58)
P score change	−1.96 (14.82)	6.67 (15.63)^a^	−9.63 (16.04)^a^	−1.25 (11.87)	−1.07 (15.37)
Pre_N2 score	38.91 (12.45)	38.02 (16.95)^c^	38.24 (14.37)	40.08 (14.47)	38.86 (14.45)
Post_N2 score	37.19 (14.74)	46.01 (12.89)^c^	31.40 (14.78)	39.85 (16.52)	39.06 (15.43)
N2 score change	−1.72 (13.15)	8.00 (12.02)^b^	−6.84 (14.11)^b^	−0.23 (8.59)	0.20 (12.88)

^a^*p* < .01, ^b^ *p* < .01, ^c^ *p* < .05

In order to examine changes in ethical thinking between the groups, we calculated a change variable for the post-conventional and N2 scores (see Table 2). The main effects of the change on the P score and on the N2 score were both significant: *F*(3, 83) = 4.26, *p* = 0.007, η = 0.13 and *F*(3,83) = 5.53, *p* = 0.002, η = 0.17, respectively. The post-hoc comparisons revealed that experiment group 2 differed significantly from control group 1 in both changes (Scheffe’s adjustment, *p*s < 0.01). Further examination revealed that in experiment group 2, 12 participants out of 23 progressed and three respectively regressed over 10 points on the P score during the ethics course. In control group 1, nine out of 18 participants regressed, and none progressed over 10 points on the P score. To compare, other groups included participants with both progression and regression leading to non-significant findings. In experiment group 1, four students out of 22 progressed and five students regressed over 10 points on the P score, whereas in control group 2, three students out of 24 progressed and five regressed, respectively.

We also examined whether the number of messages delivered by participants correlated with the change in moral reasoning scores. Bivariate correlations were all insignificant, the change on the N2 score yielding the highest correlation, *r*(45) = .19. Thus, the number of personal messages was not related to changes in moral reasoning scores.

## Discussion

The aim of the present study was to develop a method of online dilemma discussion for professional ethics education and investigate its impact on moral reasoning development. Group discussions are common in online learning, providing a new context for classic dilemma discussions, and thus deserves further study. For this purpose, an intervention study with a quasi-experimental design was designed and carried out in a graduate programme for health and social care practitioners. Participants in the intervention represented nurses, public health nurses and social welfare workers in early middle adulthood and had worked approximately for ten years in the field. Because the study took place in the context of further education, it was expected that students with a wealth of work experience would benefit from the use of real-life dilemmas as stimuli for discussions.

The participants’ average P scores on the pre-test were around 40, which are comparable with average P scores reported for undergraduate students, but lower than those reported for graduate students (Bebeau and Thoma 2003). Thus, there was substantial potential for growth in moral reasoning in all groups, that however resulted in different outcomes. Experiment group 1 did not show improvement, neither on the P score nor the N2 score; whereas, experiment group 2 did so, after rearrangements in the intervention design. The control groups did not show improvement on either score. In contrast, the scores of control group 2, consisting of business administration students, dropped significantly when compared with experiment group 1. To conclude, the present findings provided some support for the hypothesis that online dilemma discussions enhance moral reasoning development. Education may also have unintended effects that can depress moral reasoning, for example through few role-taking opportunities (Helkama et al. 2003; King and Mayhew 2002) or curricular contents (Mayhew and King 2008). Business administration students had focused on economic and social decision making in their first-term studies, which might have caused individual shifts towards personal interests and maintaining-norms thinking in the moral domain.

When we learned of non-significant findings for experiment group 1, we explored different explanations for the poor outcome of the intervention and concluded that it might be due to the coincidental group members’ homogeneity in moral reasoning. In other words, students at the same level may consolidate thinking patterns of each other rather than encourage the adoption of new ones. In order to prevent this potential inhibiting factor for the subsequent experiment group 2, student groups were composed to maximise differences in initial scores, in addition to mixing professional backgrounds. They were arranged in ways that each group would include a mixture of students with the preference of post-conventional thinking, maintaining-norms thinking and personal-interests thinking. This arrangement resulted in positive outcomes, when approximately half of the students progressed over 10 points on the P score during the ethics course. The wide array of moral schemas in group discussions may provide improved opportunities for so-called socio-cognitive conflicts that are situations in which different people represent different viewpoints, making it difficult for one to comply with others’ opinions (Buchs et al. 2004; Doise and Mugny 1984), thus advancing moral problem solving. They can also provide scaffolding and enable one to model and monitor a more-advanced peer’s performance as suggested by Vygotsky’s (1978) zone of proximal development. This improves learning and makes it possible for students to perform better in a group than alone.

To further explain differences between the experiment groups, their self-assessments of learning reported previously (Juujärvi 2018) might be useful. Students of experiment group 1 gave lower assessments than experiment group 2 for the item “Reflecting on work-related problems has advanced my learning”. They were also less active in discussions, sending a fewer number of messages, even though the difference did not reach statistical significance. We also observed that some participants reported an ethical dilemma that they have faced earlier, during their early careers. The dilemmas might have been personally significant at that time but seemed simple from the recent professional perspective.

These observations above imply that the activation of moral schemas is highly contextual depending on the dilemma at hand (Roche and Thoma 2017). It follows that real-life dilemmas, without explicit or implicit reference to society-level issues, do not challenge students’ default schemas enough and fail to boost high-level moral reasoning. Present findings thus suggest that real-life dilemmas of own personal choice would not be optimal for advancing growth in moral reasoning. On the other hand, the use of hypothetical dilemmas does not always guarantee positive outcomes either (Serodio et al. 2016). The use of real-life dilemmas in this study however brought about other positive outcomes, while students had become more aware of their attitudes and values, and they learned to solve ethical problems in their field by bringing personal experiences and theoretical knowledge together (Juujärvi 2018). From the perspective of professional expertise, the integration of tacit practical knowledge with theoretical concepts is of utmost importance (Tynjälä et al. 2020). Professionals need to be able articulate and communicate their values and ethical convictions to other people in adequate ways, for example when they feel morally compelled to advocate clients’ rights in their organisations or in the media.

To conclude, the present findings suggest that the quality of discussion plays a critical role in advancing students’ moral reasoning. It can be enhanced by the appropriate group structure, guided choice of dilemmas, and positive group climate. In the study conditions, we had the luxury to arrange groups based on their scorings on the pre-test, which is ruled out in ordinary conditions, unless the DIT is an integral part of the curriculum (Bebeau 2002). The findings underscore the importance of securing students’ exposure to higher schema thinking. Small-group discussions alone may not be sufficient to enhance students’ moral reasoning, but complementary methods are needed. Plenary discussions and debates on current issues, arranged synchronously or face-to-face, enlarge the possibilities to hear sophisticated arguments from others. It could also be useful to introduce difficult ethical cases and to directly teach certain logical and philosophical perspectives that can be applied in ethical problem solving (Penn William Jr 1990; Rest and Narváez 1994).

It seemed obvious that dilemma discussions may have enhanced other components required in ethical decision making: ethical sensitivity, motivation and implementation skills (Rest and Narváez 1994). These components are equally important goals for interventions in ethics education, but at the moment, there is a lack of appropriate methods to measure students’ advancement with them. The DIT is also not appropriate for measuring fine-grained but important changes in everyday moral reasoning, such as the improved recognition and application of relevant ethical concepts, which is a cornerstone for quality ethical decision making. The construction of valid measures is highly recommended, especially for the needs of longitudinal and intervention studies.

This study has some limitations. First, because we were not able to arrange a regular in-person ethics course as a control group, we could not compare the effect of online and in-person discussions, which would be of interest. However, this concern might be outdated, because various forms of online learning have superseded in-person group discussions as a consequence of the Covid-19 pandemic. Second, because the experiment groups were consecutive, the experiences with the first group might have carried effects to the second group. The first author acted as an interventionist in both groups, and she might be more skilled with the latter group. Moreover, because she was aware of the disappointing results with the first group, she was also highly motivated to improve the quality of the intervention. The effect of the interventionist as a group instructor cannot be distinguished from effects of other group members. Taken together, intervention studies in natural settings are hard to realise, and there will always be several variables out of the researchers’ control.

This study adds to our understanding of moral reasoning in mature adulthood, while the majority of studies has been limited to college years. Our conclusions are consistent with the view that moral development continues across the lifespan when people are confronted with new challenges and responsibilities for others’ wellbeing, and it can be supported by means of education (Rest and Narváez 1994). Adults are eager to learn new ways of thinking and problem solving, and therefore, they appreciate the ethics courses in further education that provide them with an opportunity of shared reflection in the midst of rushed working lives.

## References

[CR1] Auger GA, Gee C (2016). Developing moral maturity: An evaluation of the media ethics course using the DIT-2. Journalism & Mass Communication Educator.

[CR2] Banks S, Williams R (2005). Accounting for ethical difficulties in social welfare work: Issues, problems and dilemmas. British Journal of Social Work.

[CR3] Bebeau MJ, Rest JJ, Narváez D (1994). Influencing moral dimensions of dental *practice*. Moral development in the professions: Psychology and applied ethics.

[CR4] Bebeau MJ (2002). The defining issues test and the four component model: Contributions to professional education. Journal of Moral Education.

[CR5] Bebeau MJ, Thoma SJ (1999). “Intermediate” concepts and the connection to moral education. Educational Psychology Review.

[CR6] Bebeau MJ, Thoma SJ (2003). *Draft guide for DIT-2. Version 3.0.* Minneapolis: Centre for the Study of ethical development.

[CR7] Boss JA (1994). The effect of community service work on the moral development of college ethics students. Journal of Moral Education.

[CR8] Buchs C, Butera F, Mugny G, Darnon C (2004). Conflict elaboration and cognitive outcomes. Theory Into Practice.

[CR9] Bunch WH (2005). Changing moral judgment in divinity students. Journal of Moral Education.

[CR10] Cain J, Smith D (2009). Increasing moral reasoning skills through online discussions. The Quarterly Review of Distance Education.

[CR11] de Casterlé BD, Izumi S, Godfrey NS, Denhaerynck K (2008). Nurses’ responses to ethical dilemmas in nursing practice: meta-analysis. Journal of Advanced Nursing.

[CR12] Doise W, Mugny G (1984). The social development of the intellect.

[CR13] Duckett LJ, Ryden MB, Rest JR, Narváez D (1994). Education for ethical nursing practice. Moral development in the professions: Psychology and applied ethics.

[CR14] Fletcher J (1966). Situation ethics. The new morality.

[CR15] Graham CR, Curtis Bonk J, Graham CR (2012). Definition, current trends, and future directions. The handbook of blended learning: Global perspectives, local designs.

[CR16] Hedayati-Mehdiabadi A, Huang WD, Eunjung Grace O (2020). Understanding students’ ethical reasoning and fallacies through asynchronous online discussion: Lessons for teaching evaluation ethics. Journal of Moral Education.

[CR17] Helkama K, Uutela A, Pohjanheimo E, Salminen S, Koponen A, Rantanen-Väntsi L (2003). Moral reasoning and values in medical school: A longitudinal study in Finland. Scandinavian Journal of Educational Research.

[CR18] Hurtado S, Mayhew MJ, Engberg ME (2012). Diversity courses and students’ moral reasoning: A model of predispositions and change. Journal of Moral Education.

[CR19] Huschle BJ (2013). Assessing learner outcomes in traditional and online medical ethics courses. Teaching Philosophy.

[CR20] ICN (International Council of Nurses). (2012). the ICN code for ethics of nurses. Geneve. http://www.old.icn.ch/who-we-are/code-of-ethics-for-nurses/. Accessed 1 Nov 2021.

[CR21] IFSW (International Federation of Social Workers). (2018). Global social work statement of ethical principles. Published 2 July 2018. https://www.ifsw.org/global-social-work-statement-of-ethical-principles/. Accessed 1 November 2021.

[CR22] Joiner R, Jones S (2003). The effects of communication medium on argumentation and the development of critical thinking. International Journal of Educational Research.

[CR23] Jonassen DH, Kwon HII (2001). Communication patterns in computer mediated versus face-to-face group problem solving. Educational Technology Research and Development.

[CR24] Juujärvi S (2006). The ethic of care development: A longitudinal study of moral reasoning among practical-nursing, social-work and law-enforcement students. Scandinavian Journal of Psychology.

[CR25] Juujärvi, Soile. 2018. Practical problem solving in enhancing ethical competences of health and social care professionals. In *Learning by developing 2.0: Case studies in theory and practice*, ed. Sanna Juvonen, Päivi Marjanen, and Tarja Meristö, 178–196. Laurea University of Applied Sciences https://urn.fi/URN:ISBN:978-951-799-502-3. Accessed 1 November 2021.

[CR26] Juujärvi S, Helkama K, Kallio EK (2020). Duties and responsibilities in adulthood: Integrating care and justice perspectives. Development of adult thinking: Perspectives from psychology, education and human resources.

[CR27] Juujärvi S, Kallunki E, Luostari H (2020). Ethical decision-making of social welfare workers in the transition of services: The ethics of care and justice perspectives. Ethics and Social Welfare.

[CR28] Juujärvi S, Pesso K (2008). Pienryhmäkeskustelu eettisen herkkyyden ja ongelmanratkaisun kehittäjänä [small-group discussion as a method of promoting ethical sensitivity and problem-solving]. Kasvatus.

[CR29] Keefer M, Ashley KD (2001). Case-based approaches to professional ethics: A systematic comparison of students’ and ethicistsä moral reasoning. Journal of Moral Education.

[CR30] King PM, Mayhew MJ (2002). Moral judgment development in higher education: Insights from the defining issues test. Journal of Moral Education.

[CR31] Kohlberg L (1984). Essays on moral development. The psychology of moral development. The nature and validity of moral stages.

[CR32] Lechhasseur, Kathleen, Chantal Caux, Stéphanie Dollé, and Alain Legault. 2018. Ethical competence: An integrative review. *Nursing Ethics* 25 (6): 694–706. 10.1177/0969733016667773 27694548

[CR33] Lies JM, Bock T, Brandenberger J, Trozzolo TA (2012). The effects of off-campus service learning on the moral reasoning of college students. Journal of Moral Education.

[CR34] Mayhew MJ, King P (2008). How curricular content and pedagogical strategies affect moral reasoning development in college students. Journal of Moral Education.

[CR35] Mayhew MJ, Pascarella ET, Trolian T, Selznick B (2015). Measurements matter: Taking the DIT-2 multiple times and college students’ moral reasoning development. Research in Higher Education.

[CR36] McLeod-Sordjan R (2014). Evaluating moral reasoning in nursing education. Nursing Ethics.

[CR37] Molloy C, McCarthy J, Tyrrell M (2016). The impact of an end-of-life healthcare ethics educational intervention. Clinical Ethics.

[CR38] Myyry L, Juujärvi S, Pesso K (2013). Change in values and moral reasoning during higher education. European Journal of Developmental Psychology.

[CR39] Narváez D, Bock T (2002). Moral schemas and tacit judgment on how the defining issues test is supported by cognitive science. Journal of Moral Education.

[CR40] Numminen OH, Leino-Kilpi H (2007). Nursing students’ ethical decision-making: A review of the literature. Nurse Education Today.

[CR41] O'Flaherty J, Gleeson J (2014). Longitudinal study of levels of moral reasoning of undergraduate students in an Irish university: The influence of contextual factors. Irish Educational Studies.

[CR42] Özçinar H (2015). Scaffolding computer-mediated discussion to enhance moral reasoning and argumentation quality in pre-service teachers. Journal of Moral Education.

[CR43] Parker III, Eugene T, Barnhardt CL, Pascarella ET, McCovin JA (2016). The impact of diversity courses on college students’ moral development. Journal of College Student Development.

[CR44] Penn William Y (1990). Teaching ethics – A direct approach. Journal of Moral Education.

[CR45] Putman SM, Ford K, Tancock S (2012). Redefining online discussions: Using participant tances to promote collaboration and cognitive engagement. International Journal of Teaching and Learning in Higher Education.

[CR46] Rest JR, Rest JJ, Narváez D (1994). Background: Theory and research. Moral development in the professions: Psychology and applied ethics.

[CR47] Rest JR, Narváez D (1994). Moral development in the professions: Psychology and applied ethics.

[CR48] Rest, James R., Darcia Narváez, Muriel J. Bebeau, and Stephen J. Thoma. 1999. Postconventional moral thinking. A neo-Kohlbergian approach. Mahwah: Erlbaum.

[CR49] Roche Cicely, Thoma Steve (2017). Insights from the defining issues test on moral reasoning competencies development in community pharmacists. American Journal of Pharmaceutical Education.

[CR50] Saat MM, Porter S, Woodbine G (2012). A longitudinal study of accounting students' ethical judgement making ability. Accounting Education.

[CR51] Schlaefli A, Rest JR, Thoma SJ (1985). Does moral education improve moral judgment? A meta-analysis of intervention studies using the defining issues test. Review of Educational Research.

[CR52] Self DJ, Olivarez M, DeWitt C, Baldwin jr. (1998). The amount of small-group case-study discussion needed to improve moral reasoning skills of medical students. Academic Medicine.

[CR53] Serodio A, Kopelman BI, Batagli PUR (2016). The promotion of medical students’ moral development: A comparison between a traditional course on bioethics and a course complemented with the Konstanz method of dilemma discussion. International Journal of Ethics Education.

[CR54] Spanjers IAE, Könings KD, Leppink J, Verstegen DML, de Jong N, Czabanowska K, van Merriënboer JJG (2015). The promised land of blended learning: Quizzes as a moderator. Educational Research Review.

[CR55] Stone JR, Chapple HS, Haddad A, Lux S, Rentmeester CA (2017). Discussion in graduate online bioethics programs. International Journal of Ethics Education.

[CR56] Thoma SJ, Killen M, Smetana J (2006). Research on the Defining Issues Test. Handbook of moral development.

[CR57] Thoma SJ (2014). Measuring moral thinking from a neo-Kohlbergian perspective. Theory and Research in Education.

[CR58] Thoma SJ, Bebeau MJ, Bolland A, Oser FK, Veugelers W (2008). The role of moral judgment in context-specific professional decision making. Getting involved: Global citizenship development and sources of moral values.

[CR59] Thoma S, Dong Y (2014). The defining issues test of moral judgment development. Behavioral Development Bulletin.

[CR60] Tynjälä P, Kallio EK, Heikkinen HLT, Kallio EK (2020). Professional expertise, integrative thinking, wisdom and phronesis. Development of adult thinking: Perspectives from psychology, education and human resources.

[CR61] Vygotsky LS (1978). Mind in society.

